# Retraction: Repurposing food molecules as a potential BACE1 inhibitor for Alzheimer's disease

**DOI:** 10.3389/fnagi.2026.1875922

**Published:** 2026-05-14

**Authors:** Frontiers Editorial Office

The journal retracts the 22 August 2022 article cited above.

Following publication, concerns were raised regarding the contributions of the authors of the article as well as an undisclosed conflict of interest between author, Ghulam Md Ashraf, and reviewer, Anas Shamsi. Our investigation, conducted in accordance with Frontiers policies, confirmed an undisclosed conflict of interest that undermined the integrity of the peer review process, and impacted peer reviewer recommendations, as well as a serious breach of our authorship policies. Post-publication assessment of the article confirmed that the article does not meet the standards of the journal and as a result, the conclusions of the article have been deemed unreliable. In adherence to the recommendations of the Committee on Publication Ethics (COPE), the article is retracted.

This retraction was approved by the Chief Executive Editor of Frontiers. The authors received a communication regarding the retraction and had a chance to respond. This communication is recorded by the publisher.

Frontiers would like to thank Elisabeth Bik and the users on PubPeer for bringing the published article to our attention.

